# Clinical and cortical similarities identified between bipolar disorder I and schizophrenia: A multivariate approach

**DOI:** 10.3389/fnhum.2022.1001692

**Published:** 2022-11-10

**Authors:** Kelly Rootes-Murdy, Jesse T. Edmond, Wenhao Jiang, Md A. Rahaman, Jiayu Chen, Nora I. Perrone-Bizzozero, Vince D. Calhoun, Theo G. M. van Erp, Stefan Ehrlich, Ingrid Agartz, Erik G. Jönsson, Ole A. Andreassen, Lars T. Westlye, Lei Wang, Godfrey D. Pearlson, David C. Glahn, Elliot Hong, Robert W. Buchanan, Peter Kochunov, Aristotle Voineskos, Anil Malhotra, Carol A. Tamminga, Jingyu Liu, Jessica A. Turner

**Affiliations:** ^1^Department of Psychology, Georgia State University, Atlanta, GA, United States; ^2^Tri-Institutional Center for Translational Research in Neuroimaging and Data Science (TReNDS), Georgia Institute of Technology, Georgia State University, Emory University, Atlanta, GA, United States; ^3^Department of Psychosomatics and Psychiatry, Medical School, Zhongda Hospital, Institute of Psychosomatics, Southeast University, Nanjing, China; ^4^Department of Neurosciences, University of New Mexico, Albuquerque, NM, United States; ^5^Clinical Translational Neuroscience Laboratory, Department of Psychiatry and Human Behavior, University of California, Irvine, Irvine, CA, United States; ^6^Center for the Neurobiology of Learning and Memory, University of California, Irvine, Irvine, CA, United States; ^7^Division of Psychological and Social Medicine and Developmental Neurosciences, Faculty of Medicine, TU Dresden, Dresden, Germany; ^8^Division of Mental Health and Addiction, Norwegian Centre for Mental Disorders Research (NORMENT), Institute of Clinical Medicine, Oslo University Hospital, University of Oslo, Oslo, Norway; ^9^Department of Clinical Neuroscience, Centre for Psychiatry Research, Karolinska Institute and Stockholm Health Care Services, Stockholm, Sweden; ^10^K. G. Jebsen Centre for Neurodevelopmental Disorders, University of Oslo, Oslo, Norway; ^11^Department of Psychiatric Research, Diakonhjemmet Hospital, Oslo, Norway; ^12^Department of Psychology, University of Oslo, Oslo, Norway; ^13^Psychiatry and Behavioral Health, Ohio State Wexner Medical Center, Columbus, OH, United States; ^14^Department of Psychiatry, Yale University, New Haven, CT, United States; ^15^Olin Neuropsychiatry Research Center, Institute of Living, Hartford Hospital, Hartford, CT, United States; ^16^Boston Children’s Hospital and Harvard Medical School, Boston, MA, United States; ^17^Department of Psychiatry, Maryland Psychiatric Research Center, University of Maryland School of Medicine, Baltimore, MD, United States; ^18^Department of Psychiatry, Centre for Addiction and Mental Health, University of Toronto, Toronto, ON, Canada; ^19^Division of Psychiatry Research, Zucker Hillside Hospital, Queens, NY, United States; ^20^Department of Psychiatry, University of Texas Southwestern Medical School, Dallas, TX, United States

**Keywords:** bipolar disorder, schizophrenia, multivariate analysis, ICA, PANSS

## Abstract

**Background:**

Structural neuroimaging studies have identified similarities in the brains of individuals diagnosed with schizophrenia (SZ) and bipolar I disorder (BP), with overlap in regions of gray matter (GM) deficits between the two disorders. Recent studies have also shown that the symptom phenotypes associated with SZ and BP may allow for a more precise categorization than the current diagnostic criteria. In this study, we sought to identify GM alterations that were unique to each disorder and whether those alterations were also related to unique symptom profiles.

**Materials and methods:**

We analyzed the GM patterns and clinical symptom presentations using independent component analysis (ICA), hierarchical clustering, and n-way biclustering in a large (*N* ∼ 3,000), merged dataset of neuroimaging data from healthy volunteers (HV), and individuals with either SZ or BP.

**Results:**

Component A showed a SZ and BP < HV GM pattern in the bilateral insula and cingulate gyrus. Component B showed a SZ and BP < HV GM pattern in the cerebellum and vermis. There were no significant differences between diagnostic groups in these components. Component C showed a SZ < HV and BP GM pattern bilaterally in the temporal poles. Hierarchical clustering of the PANSS scores and the ICA components did not yield new subgroups. N-way biclustering identified three unique subgroups of individuals within the sample that mapped onto different combinations of ICA components and symptom profiles categorized by the PANSS but no distinct diagnostic group differences.

**Conclusion:**

These multivariate results show that diagnostic boundaries are not clearly related to structural differences or distinct symptom profiles. Our findings add support that (1) BP tend to have less severe symptom profiles when compared to SZ on the PANSS without a clear distinction, and (2) all the gray matter alterations follow the pattern of SZ < BP < HV without a clear distinction between SZ and BP.

## Introduction

Schizophrenia (SZ) and bipolar disorder (BP) are characterized by biological and clinical heterogeneity. Neuroimaging studies have found stable and replicable brain structural alterations associated with one or the other disorder [e.g., schizophrenia (Aine et al., 2017; Kochunov et al., 2019)]. There are also gray matter abnormalities related to specific symptom profiles such as psychosis (Meda et al., 2015; Clementz et al., 2016) or catatonia states (Hirjak et al., 2020), and to duration of illness instead of diagnosis, as well as confounds of medication(s) (Hartberg et al., 2015; Vita et al., 2015; Jørgensen et al., 2016; di Sero et al., 2019; Barth et al., 2020), or even structural patterns that cross diagnostic lines (Andreassen et al., 2013; Yao et al., 2017; Ruderfer et al., 2018; Kochunov et al., 2022). Untangling these unique structural features and related symptoms profiles from one another may allow us to find clinically meaningful subgroups within each disorder that may aid in the development of more precise diagnostic and treatment options.

Schizophrenia and BP are severe, heritable, and most importantly, debilitating mental illnesses both categorized by cognitive impairments, affective symptoms, and behavioral dysfunction (American Psychiatric Association, 2013). Structural neuroimaging studies have also identified similarities in the brain correlates of SZ and BP, with overlap in regions of gray matter (GM) deficits between the two disorders (Doan et al., 2017; Schwarz et al., 2019; Sorella et al., 2019; Lee D.-K. et al., 2020; Cheon et al., 2022). Accumulating evidence suggests that these two disorders may be better described along a continuum, not as two distinct disorders, of varying cognitive deficits and psychosis (Jabben et al., 2010; Hill et al., 2013). Examining SZ and BP together, as opposed to separately, may allow for the emergence of unique subgroups that may have been previously masked by diagnostic categories. Clementz et al. (2016) identified three distinct homogeneous subtypes based on a clinical profile of cognition and sensorimotor responses to functional tasks, rather than diagnostic boundaries, when SZ and BP with psychosis samples were combined (Clementz et al., 2016). This approach, and specifically these biomarkers have shown to replicate over time (Clementz et al., 2022) and have identified more precise representations of the symptom profiles associated with SZ and BP (Hudgens-Haney et al., 2020). We sought to expand upon these results through a multivariate approach and a larger dataset (*N* ∼ 3,000) of both individuals with schizophrenia and individuals with bipolar disorder I with psychosis.

While the heterogeneity between patients is substantial (Alnæs et al., 2019; Wolfers et al., 2021), schizophrenia has been associated with regional gray matter reductions throughout the cortex, analyzed either voxel by voxel or region by region (Honea et al., 2005; van Erp et al., 2018; Rootes-Murdy et al., 2021). Source-based morphometry (SBM), a multivariate approach, has identified covarying patterns of lower gray matter concentrations in the salience network, default mode network, insula-medial prefrontal cortex (MPFC), and the cerebellum (Turner et al., 2012; Ivleva et al., 2013; Gupta et al., 2015). One of the largest regional gray matter concentration effects was noted in the superior temporal gyrus (Gupta et al., 2015) and the cerebellum (Moberget et al., 2018). However, higher gray matter concentrations have been identified in the cerebellum (Gupta et al., 2015) and bilaterally in both the pre- and postcentral gyri (Mennigen et al., 2019). Regardless of the few discrepancies, there are clear patterns and structures with GM deficiencies in individuals with SZ. Similarly, gray matter analyses in BP have shown volumetric reductions throughout the cortex, with smaller effect sizes compared to SZ, however, these studies viewed BP in a general manner and did not specify bipolar subtype, or whether participants had psychosis (Murray et al., 2004; Bora, 2015; de Zwarte et al., 2019). Therefore, it remains unclear whether these two disorders are representations of discrete categories or if they fall along a spectrum of cognitive, affective, and behavioral disturbances.

In this study, we took a cross-disorder approach motivated by the overlapping symptoms and structural patterns observed in both schizophrenia and bipolar disorder. However, given the symptom variation within each disorder, we hypothesized that there may be subclusters within and across each disorder, that may help distinguish the underlying neural networks involved in each disorder. For example, delusions in bipolar disorder, were associated with gray matter reductions largely in the frontal cortex and amygdala and SZ showed similar patterns with additional contributions from subcortical regions (Rootes-Murdy et al., 2022). Within individuals with SZ, a symptom phenotype consisting of higher delusional symptoms, suspiciousness, hallucinations, and anxiety [measured from the Positive and Negative Syndrome Scale (PANSS); (Kay et al., 1987)] was associated with a covarying pattern of lower GM concentration in inferior temporal gyri and fusiform gyri and higher GM concentration in the sensorimotor cortex (Mennigen et al., 2019). A similar analysis in BP showed a symptom phenotype of mood symptoms (anxiety, depression, and guilt) was associated with lower GM concentration in the right middle/superior temporal gyrus (Jiang et al., 2020). The use of a large, cross-diagnostic sample may allow for examination of covarying gray matter patterns that are associated with these two disorders and with specific symptom presentations.

Structural studies in schizophrenia and bipolar disorder report inconsistencies on the patterns that are unique to each disorder, and how the shared symptoms between disorders relate to the structural deficits identified within the disorders. For this study, we sought to examine the differences and similarities in gray matter and symptom profiles between schizophrenia and bipolar disorder in a three-pronged approach. To compare the patterns of gray matter variation in a large, combined sample of schizophrenia, bipolar disorder, and healthy volunteers we used a multivariate data-driven approach, independent component analysis (ICA). Next, we used a hierarchical cluster analysis of psychosis subscales (PANSS positive, negative, and general) to identify patterns in the symptom presentation of schizophrenia and bipolar disorder and examine the groupings in symptom presentation. We also used hierarchical cluster analysis to identify pattern groupings in the gray matter concentration across participants using the ICA gray matter loading coefficients. Finally, we applied an N-way biclustering analysis (Rahaman et al., 2020) using both the PANSS subscales and the ICA loading coefficients to identify possible subgroups within and across each disorder that combine unique symptom and gray matter profiles.

## Materials and methods

### Participants

This study included data from 1,217 individuals diagnosed with schizophrenia, 301 individuals diagnosed with bipolar disorder I, and 1,543 unrelated healthy volunteers from the following datasets, many previously described in the literature; the Functional Imaging Biomedical Information Research Network study (FBIRN 3; multiple sites in the USA) (Potkin et al., 2009), the Center of Biomedical Research Excellence study (COBRE; Albuquerque, NM, USA) (Gupta et al., 2015), the Bipolar and Schizophrenia Network for Intermediate Phenotypes 1 study (B-SNIP 1; multiple sites in the USA) (Meda et al., 2015), the MIND Clinical Imaging Consortium study (MCIC; Albuquerque, NM, USA) (Gollub et al., 2013), the Northwestern University Schizophrenia Data study (NW; Chicago, IL, USA) (Wang et al., 2013), the Human Brain Informatics (HUBIN; Stockholm, Sweden) (Hall et al., 2002), Thematic Organized Psychosis [(TOP) research; Oslo, Norway] (Ringen et al., 2008; Rimol et al., 2012), Olin (Olin Center for Neuropsychiatric Research) (Yao et al., 2017), the Maryland Psychiatric Research Center (MPRC, Baltimore, MD, USA) (Kochunov et al., 2016, 2017), and from the Centre for Addiction and Mental Health (CAMH, Toronto, Canada) (Hawco et al., 2019). A diagnosis of SZ was confirmed by the Structured Clinical Interview for Diagnosis (SCID) for Diagnostic and Statistical Manual of Mental Health 4th Edition (DSM-IV or DSM-IV TR) as part of each study site’s protocol. For this study, participants with a diagnosis of SZ or schizophreniform were included. All data were collected under approval of local institutional review boards and all participants provided informed consent. The original study designs are described in the previous publications (cited above). Scanning information for each site can be found in Table 1. Participant demographic information including age, sex, diagnosis, and PANSS scores are included in Table 2.

**TABLE 1:** Scanning and site information for each dataset.

Study	Size	Sites	Scanner (T)	Sequence	Voxel size (mm)	Orientation
BSNIP 1	773	5	GE Signa (3)	MPRAGE	1 × 1 × 1	Sagittal
			Philips Achieva (3)	IR-SPGR		
			Siemens Allegra (3)			
			Siemens Trio (3)			
			GE Signa HDxt (3)			
			Siemens Trio (3)			
CAMH	356	3	Siemens Trim Trio (3)	Grad Echo	1 × 1 × 1	
			GE Signa (3)	Grad Echo	1 × 1 × 1	
COBRE	148	1	Siemens Tim Trio (3)	MPRAGE	1 × 1 × 1	Sagittal
FBIRN3	343	8	Siemens Tim Trio (3)	MPRAGE	1.1 × 0.9 × 1.2	Sagittal
HUBIN	158	1	GE Signa (1.5)	SPGR	1 × 1 × 1	Coronal
MCIC	210	4	Siemens (1.5)	Grad Echo	0.625 × 0.625 × 1.5	Coronal
			GE Signa (1.5)	Grad Echo	0.664 × 0.664 × 1.6	
			Siemens Trio (3) Siemens (1.5)	MPRAGE	0.625 × 0.625 × 1.5	
				Grad Echo	0.625 × 0.625 × 1.5	
MPRC	389		Siemens Trio (3)	Grad Echo	1.7 × 1.7 × 3	Axial
			Allegra (3)		1.7 × 1.7 × 4	Axial
NW	136	1	Siemens (1.5)	MPRAGE	1 × 1 × 1	
Olin	159	1	Siemens Allegra (3)	Grad Echo	3.75 × 3.75 × 4	Ascending
TOP	387	1	Siemens (1.5)	MPRAGE	1.33 × 0.94 × 1	Sagittal

All scanning parameter information was obtained from the original study publications.

**TABLE 2:** Demographic information across sites.

	BSNIP	CAMH	COBRE	FBIRN	HUBIN	MCIC	MPRC	NW	Olin	TOP	Total
Total participants	774	356	148	343	158	210	389	136	159	387	3060
Age in years	35.62 (12.41)	32.05 (10.34)	35.51 (11.86)	38.36 (11.32)	41.65 (8.61)	33.08 (10.84)	35.69 (14.20)	32.72 (13.16)	32.74 (11.90)	33.41 (9.63)	35.41 (21.12)
Diagnosis											
BD	191	0	0	0	0	0	0	0	0	109	300
SZ	248	208	71	175	92	93	124	70	40	96	1217
HV	335	148	77	168	66	117	265	66	119	182	1543
Males (%)	376 (48.6%)	222 (62.4%)	111 (75%)	251 (73.2%)	107 (67.7%)	141 (67.1%)	205 (52.7%)	87 (64%)	87 (54.7%)	202 (52.2%)	1789 (58.5%)
Duration of illness in years	__	__	16.0 ± 12.0	18.0 ± 11.8	__	12.32 ± 10.5	__	13.09 ± 12.4	__	7.1 ± 6.3	14.27 ± 11.2
CPZ equivalent in mg/d	__	__	516.3 ± 1095.4	542.3 ± 1271.4	__	511.70 ± 721.9	__	__	__	__	653 ± 1094.9
Total PANSS scores	418	0	68	172	0	0	0	0	2	200	860
Positive subscale	15.12 (5.44)	__	15.18 (4.96)	15.37 (5.00)	__	__	__	__	11.00 (1.41)	12.11 (5.12)	14.47 (5.39)
Negative subscale	14.44 (5.64)	__	14.71 (4.62)	14.47 (5.59)	__	__	__	__	19.50 (13.44)	12.35 (5.91)	13.99 (5.70)
General subscale	30.71 (8.55)	__	29.93 (8.68)	28.63 (87.35)	__	__	__	__	30.00 (5.66)	28.28 (7.69)	29.66 (8.19)
PANSS total score	60.27 (16.70)	__	59.81 (14.40)	58.50 (14.85)	__	__	__	__	60.50 (17.68)	52.75 (16.08)	58.12 (16.28)

All values are means unless otherwise noted, SD in parenthesis. SD, standard deviation; BD, bipolar disorder; SZ, schizophrenia; HV, healthy volunteers; AP, antipsychotic medication; CPZ, chlorpromazine equivalent; PANSS, positive and negative symptom scale. Duration of illness was calculated as follows: COBRE, calculated by subtracting age at first psychiatric illness/symptoms from current age; FBIRN, calculated by subtracting age at first psychotic onset from current age; NW, DOI listed; MCIC, LENGTH_OF_ILLNESS variable which was based on diagnosis/treatment/onset through different algorithm.

### MRI acquisition and preprocessing

T1-weighted structural MRI images were acquired from various scanners with information further detailed in the original studies (see Table 1). All T1-weighted images used the following preprocessing protocol for data harmonization. Images were co-registered and normalized to the standard Montreal Neurological Institute (MNI) template using a 12-parameter affine model, resliced to a voxel size of 2 mm × 2 mm × 2 mm and segmented into gray matter, white matter, and cerebro-spinal fluid using Statistical Parametric Mapping 12 (SPM12).^1^ Individual images were correlated with the group-generated gray matter template, and images with low correlations with the template (*r* < 0.87) were removed as outliers in keeping with the standards of previous studies (Turner et al., 2012; Gupta et al., 2015). The remaining images were smoothed at 8 mm FWHM prior to analyses. These preprocessing steps resulted in a total of 3,018 gray matter concentration images (1,217 individuals diagnosed with schizophrenia, 301 individuals diagnosed with bipolar disorder I, and 1,543 unrelated healthy volunteers).

### Independent component analysis

We utilized the source-based morphometry (SBM) module of the GIFT Toolbox^2^ to perform independent component analysis (ICA) (Xu et al., 2009; Gupta et al., 2019). SBM identifies patterns which covary among the participants. This approach decomposes the gray matter images of the dataset into linear combinations of gray matter patterns or components. SBM is a linear model with the sum of component maps and participant loadings making up the input segmentation maps. This technique results in components (or patterns) of gray matter which covary across participants. The contribution of a component for each participant, or the individual loading coefficient, indicates that that pattern of gray matter variation is specifically weighted for that individual (Xu et al., 2009).

The minimum description length (MDL) algorithm (Rissanen, 2018) estimated the number of components for the gray matter structural data to be 44. To ensure stability, the infomax ICA algorithm was applied with 10 runs and ICASSO (Himberg et al., 2004) determined the most stable run and number of components. High component stability was achieved for all but two components (*N* = 42).

Of the 44 components estimated in the ICA, the components with the highest variance percentages (>5%) were selected for the main analyses (4 total). First, we applied a linear mixed model (LMM) correction to the loading coefficients, including age and sex as fixed effects, and site (29 in total) as a random effect. The corrected loading coefficients were used in an ANOVA model as the dependent variables, with diagnostic group as a factor (healthy volunteers, bipolar disorder, and schizophrenia). Statistical results for all group comparisons (HV v BP; HV v SZ; BP v SZ) were thresholded at α < 0.0125 with Bonferroni (*p* = 0.05/4) correction. Secondary ANOVA models for additional analyses are described below. For the secondary analyses, statistical results for all group comparisons (HV v BP; HV v SZ; BP v SZ) were thresholded at α < 0.00125 with Bonferroni (*p* = 0.05/40) correction. All models were completed using R v3.6.2 (R Core Team, 2014).

### Assessments

The Positive and Negative Syndrome Scale (PANSS) is the gold-standard symptom assessment for psychotic disorders (Kay et al., 1987) and one of the most commonly used questionnaires for symptom assessment in SZ over the last 2 weeks, and given the symptom overlap the PANSS has also been adopted to assess BP symptomatology (Kaltenboeck et al., 2016). Briefly, the PANSS allows for assessment of dimension specific abnormalities across positive, negative, and general symptoms. The positive symptom subscale includes seven items: delusions, conceptual disorganization, hallucinations, excitement, grandiosity, suspiciousness, and hostility (range: 7–49). The negative symptom subscale includes seven items: blunted affect, emotional withdrawal, poor rapport, passive/apathetic social withdrawal, difficulty in abstract thinking, lack of spontaneity and flow of conversation, and stereotyped thinking (range: 7–49). The general symptom subscale includes 16 items: somatic concerns, anxiety, guilt feelings, tension, mannerisms and posturing, motor retardation, uncooperativeness, unusual thought content, disorientation, poor attention, lack of judgment and insight, disturbance of volition, poor impulse control, preoccupation, and active social avoidance (range: 16–112). The PANSS was administered at six study sites, totaling 860 individuals with a completed PANSS (both SZ and BP diagnoses were included). For the purposes of this study, the PANSS subscales (positive, negative, and general psychopathology) were individually totaled and used for subsequent hierarchical cluster analyses and bicluster analyses (see below).

### Hierarchical cluster analysis

Using individuals with PANSS scores (*N* = 860), we completed spectral clustering on the three subscales of the PANSS, which informed the number of eigenvalues. In brief, spectral clustering uses eigenvalues of the similarity matrix of the data to perform dimensionality reduction before clustering in the reduced space (von Luxburg, 2007). This analysis was completed using the normalized symmetric Laplacian matrix in MATLAB 2020b with the “*spectralcluster*” function (Ng et al., 2001). The number of eigenvalues approximating to or equaling zero was six and therefore, the three subscale scores were used to create six unique clusters. Using *k* = 6, we conducted a hierarchical cluster analysis with the PANSS subscales. A MANCOVA (FDR *p* corrected) was used to examine the differences in the loading coefficients from the ICA components between each of the resulting clusters of PANSS subscale scores.

The same clustering process was completed with all the loading coefficient values from the ICA (*N* = 44 components) on the same subset of individuals (*N* = 860). A hierarchical cluster analysis was utilized with *k* = 4 based on the spectral clustering estimate. Once again, a MANCOVA (FDR *p* corrected) was used to examine the differences in the PANSS subscale scores between each of the resulting clusters of loading coefficients.

### N-way biclustering

Biclustering is a data mining technique that allows for pattern detection in large or high dimensional data. The n-way biclustering algorithm utilized in this study has been more completely described previously in the literature (Rahaman et al., 2020). We utilized the NBIC module of the GIFT Toolbox^3^ to perform n-way biclustering. The algorithm uses a depth-first search (DFS) technique to explore the data matrix (subjects by measurements) with the goal of identifying submatrices with homogeneity in the pre-selected columns. It is not a requirement of the algorithm for the submatrices to involve the entire matrix and therefore, the resulting groupings may only involve a subset of the sample. In the data matrix for this study, the columns were composed of both the PANSS subscale scores and the loading coefficients of 44 ICA components while participants were listed in the rows, resulting in an 860 × 47 matrix.

## Results

### Independent component analysis results

**FIGURE 1:**
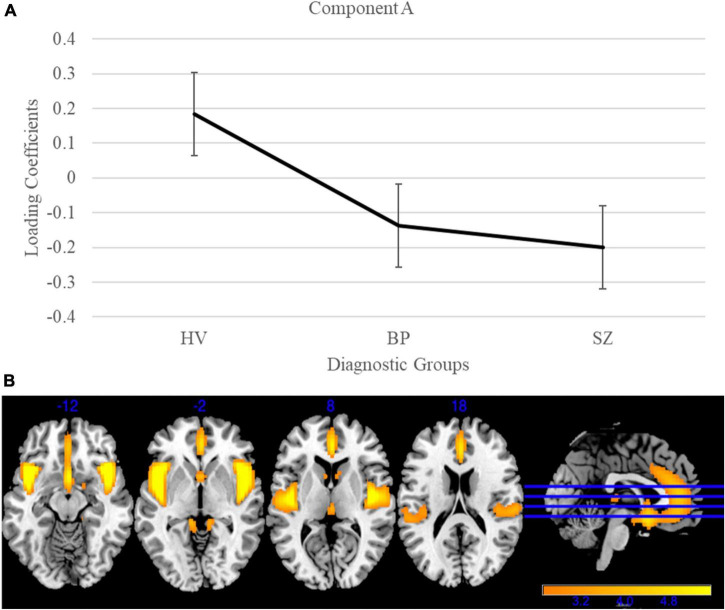
Gray matter group differences in independent component analysis (ICA) results for healthy volunteers, individuals with schizophrenia, and individuals with bipolar I disorder. **(A)** Group effects of diagnosis on loading coefficients for Component A. Schizophrenia (SZ) and bipolar disorder (BP) had significantly less gray matter when compared to healthy volunteers but did not differ significantly from each other. **(B)** Component A showed less gray matter concentration in the bilateral insula and cingulate gyrus in individuals with SZ and individuals with BP compared to healthy volunteers. Images are thresholded at 2.5.

**FIGURE 2:**
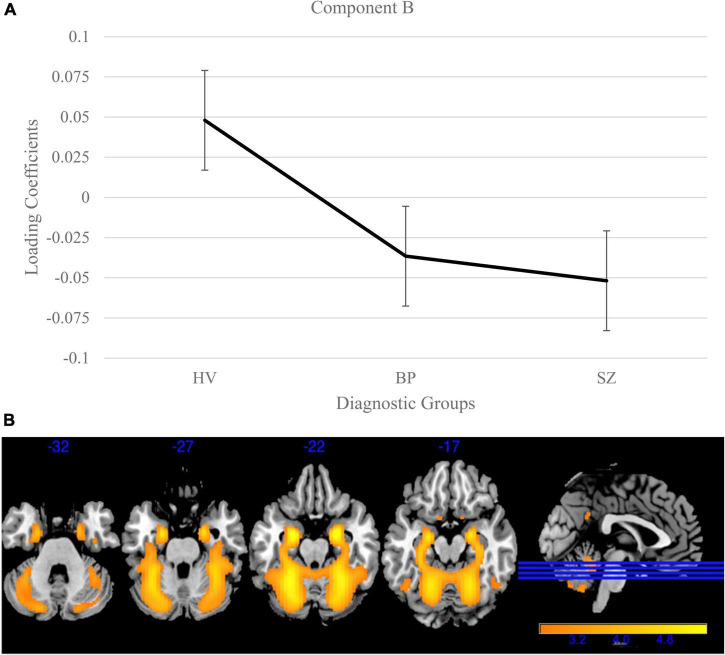
Cerebellum and vermis gray matter group differences in independent component analysis (ICA) results for healthy volunteers, individuals with schizophrenia, and individuals with bipolar I disorder. **(A)** Group effects of diagnosis on loading coefficients for Component B. Schizophrenia (SZ) and bipolar disorder (BP) had significantly less gray matter compared to healthy volunteers but did not significantly differ from each other. **(B)** Component B showed less gray matter concentration in the cerebellum and vermis in individuals with SZ and individuals with BP compared to healthy volunteers. Images thresholded at 2.5.

Gray matter clustering approach with ICA yielded 44 components. Of the 44 components, there were four components identified with high variance (>5%). From those four components, two (Components A and B) showed significant group differences; see Figures 1, 2 for more details. Component A identified a pattern of covarying gray matter bilaterally in the insula and cingulate gyrus and showed when comparing HV to both diagnostic groups [*F*(2, 3056) = 115.42, *p* < 0.001]. A Bonferroni’s test for multiple comparisons (0.05/4) showed significant differences on the Component A loading coefficients between HV and SZ [*t*(2443.73) = 14.47, *p* = 1.28E-45] and BP [*t*(1840) = 7.90, *p* < 4.78E-15]. There were no significant differences on the loading coefficients between SZ and BP identified in this component [*t*(1515) = 1.35, *p* = 0.173]. Component B also showed more gray matter concentration in the cerebellar hemispheres and vermis when comparing HV to both diagnostic groups [*F*(2, 3056) = 16.14, *p* < 0.001]. Bonferroni’s test for multiple comparisons found significant differences on the Component B loading coefficients between HV and SZ [*t*(2757) = 5.53, *p* = 3.59E-8] and BP [*t*(1840) = 2.97, *p* = 0.003]. Again, there were no significant differences between SZ and BP [*t*(1515), *p* = 0.636].

**FIGURE 3:**
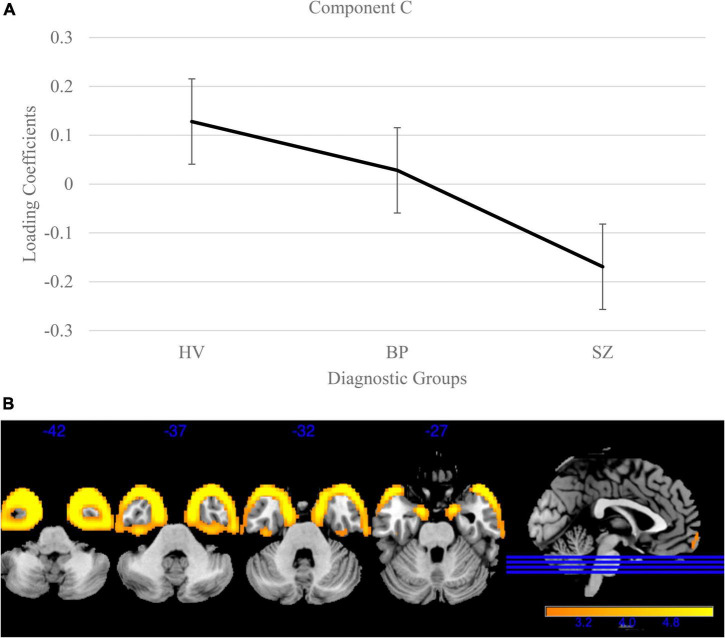
Temporal pole gray matter group differences in independent component analysis (ICA) results for healthy volunteers, individuals with schizophrenia, and individuals with bipolar I disorder. **(A)** Group effects of diagnosis on loading coefficients. Individuals with schizophrenia (SZ) had significantly less gray matter compared to individuals with bipolar disorder (BP) and healthy volunteers. Individuals with BP did not differ significantly from healthy volunteers. **(B)** Component C showed less gray matter concentration in the bilateral temporal poles in individuals with SZ compared to individuals with BP and healthy volunteers. Individuals with BP did not significantly differ from healthy volunteers. Images are thresholded at 2.5.

Next, the remaining forty loading coefficients (with low variances) were compared for group differences [Bonferroni corrected for multiple comparisons (0.05/40)] using the same models as above. One component, Component C (variance = 0.62%; Figure 3), showed a significant group effect [*F*(2, 3056) = 48.15, *p* < 0.001] and Bonferroni’s test found significant difference on the Component C loading coefficients between SZ and BP [*t*(1515) = 3.95, *p* = 8.20E-5]. This component identified a pattern of lower gray matter bilaterally in the temporal poles, with lower loading coefficients in SZ compared to BP (*p* < 0.001) and schizophrenia and healthy volunteers [*t*(2757) = 9.78, *p* < 3.06E-22]. Loading coefficients between individuals with bipolar disorder and healthy volunteers were similar for this component [*t*(1840) = 1.97, *p* = 0.05]. Component C is the only component that identified a distinction between SZ and BP.

There were 17 additional components that showed significant group effects. See Table 3 for the component numbers, their spatial map peaks, and the statistics regarding the group effect size. See Supplementary Table 1 for a list of all remaining components that did not show significant group differences.

**TABLE 3:** Significant diagnostic group differences in the independent component analysis (ICA) components.

Direction		#	Peak location	Cohen’s *F*	*P* value
HV > both	HV > SZ	8	Brainstem	0.17	7.43E-25
	HV > BP			0.06	1.58E-05
	HV > SZ	A	Insula/cingulate gyrus	0.27	4.46E-47
	HV > BP			0.14	2.44E-13
	HV > SZ	24	Medial prefrontal cortex	0.21	8.02E-06
	HV > BP			0.12	3.63E-11
	HV > SZ	26	Temporal pole	0.11	3.22E-08
	HV > BP			0.07	0.00104
	HV > SZ	27	Temporal inferior gyrus/occipital gyrus	0.06	1.52E-03
	HV > BP			0.04	4.62E-02
	HV > SZ	B	cerebellum	0.1	1.21E-07
	HV > BP			0.05	0.014
HV > SZ		5	Cerebellum/vermis	0.12	3.06E-10
		7	caudate	0.08	7.02E-05
		10	Calcarine/Crus I	0.09	5.14E-06
		18	Crus II	0.12	4.29E-09
		20	Calcarine/occipital gyrus	0.09	3.06E-05
		28	Rectus/cerebellum/fusiform gyrus	0.16	1.06E-17
		30	Insula/Rolandic operculum	0.13	1.18E-11
		C	Fusiform gyrus/temporal pole	0.18	7.76E-22
		33	Calcarine/frontal middle gyrus	0.08	1.17E-04
		34	Supplemental motor region	0.07	3.70E-04
		35	Cerebellum/parahippocampal gyrus	0.08	1.30E-04
		36	Parietal superior gyrus	0.09	1.84E-06
BP > SZ		C	Fusiform gyrus/temporal pole	0.07	7.76E-22

SZ, individuals with schizophrenia; BP, individuals with bipolar disorder; HV, healthy volunteers. Cohen’s F and p values reported from Bonferroni corrected post hoc analyses. # Component number.

### Hierarchical clustering results

**FIGURE 4:**
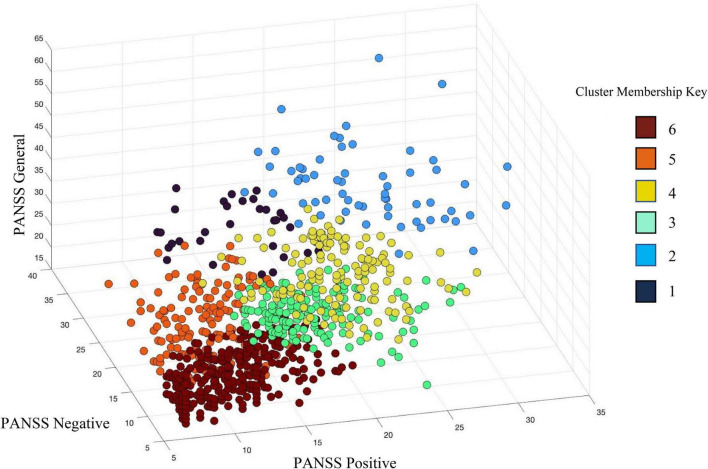
Hierarchical clustering of PANSS subscale scores identified six (6) clusters across individuals with schizophrenia (SZ) and individuals with bipolar disorder (BP). PANSS positive scores are plotted on the X-axis, PANSS negative scores on the Y-axis, and PANSS general psychopathology scores are on the Z-axis. See legend for cluster membership key.

Schizophrenia had more severe symptoms in the PANSS positive [*t*(675.649) = 11.02, *p* = 4.40E-26], PANSS negative [*t*(819.79) = 13.45, *p* = 2.10E-37], and PANSS general [*t*(658.72) = 6.16, *p* = 1.29E-9] than BP. There was also a significant site effect (*N* = 16) for all PANSS subscales; PANSS positive [*F*(15,844) = 6.266, *p* = 1.081E-12], PANSS negative [*F*(15,844) = 3.28, *p* = −2.4E-5], and PANSS general [*F*(15,844) = 7.940, *p* = 5.921E-17]. In the hierarchical clustering analysis of the PANSS scores, while each cluster had participants with either SZ or BP diagnosis, there was a significant difference in diagnostic group membership across the six clusters [χ^2^(5) = 86.68, *p* < 0.001]. Cluster 6, characterized by lowest PANSS subscale scores had a significantly higher percentage of BP (53.1% of the cluster) than did any other cluster. Cluster 1, in contrast, was characterized by the highest PANSS negative, and slightly above average PANSS positive and general subscale scores, had only two individuals with BP (6.06%). See Table 4 and Figure 4 for more information on cluster membership, average symptoms, and diagnosis by cluster. See Supplementary Figure 2 for the breakdown of the three PANSS subscales by each cluster. The clusters’ membership was used as a grouping factor in a MANCOVA on all 44 components’ loading coefficients (FDR *p* corrected). Cluster 2 had less gray matter in Component B [*F*(5,854) = 3.44, *p* = 0.004] than Cluster 3 and Cluster 6 but neither association passed FDR correction (*p* = 0.008 and *p* = 0.007, respectively). No other between groups association of cluster membership and loading coefficients passed FDR correction (all *p* values > 0.005).

**TABLE 4:** PANSS subscale spread across clusters.

	*N*	PANSS positive	PANSS negative	PANSS general	PANSS total	Age	BP (%)
CLUSTER 6	309	10.62 ± 3.00	9.54 ± 2.34	22.81 ± 3.73	42.97 ± 6.75	35.66 ± 12.00	164 (53.07%)
CLUSTER 5	151	10.74 ± 2.52	17.03 ± 3.55	26.51 ± 5.59	54.28 ± 7.60	34.44 ± 11.65	43 (28.48%)
CLUSTER 1	33	14.91 ± 3.20	26.48 ± 2.74	35.85 ± 4.67	77.24 ± 5.85	27.27 ± 7.99	2 (6.06%)
CLUSTER 3	159	17.48 ± 3.42	13.11 ± 3.08	30.70 ± 2.38	61.28 ± 5.02	36.30 ± 12.05	37 (23.27%)
CLUSTER 4	146	19.21 ± 3.15	14.19 ± 3.78	38.09 ± 3.32	71.49 ± 6.76	36.36 ± 11.69	40 (27.39%)
CLUSTER 2	62	23.56 ± 4.25	23.90 ± 5.47	45.71 ± 5.22	93.18 ± 8.19	34.05 ± 11.83	8 (12.90%)
TOTAL	860	14.47 ± 5.39	13.76 ± 5.59	29.66 ± 8.19	58.12 ± 16.28	35.25 ± 11.86	300 (38.37%)

PANSS subscale scores for each of the hierarchical cluster membership. All scores and ages are presented as mean ± standard deviation unless otherwise noted. BP, bipolar disorder. There was no significant associations between the PANSS clusters and the ICA components.

**FIGURE 5:**
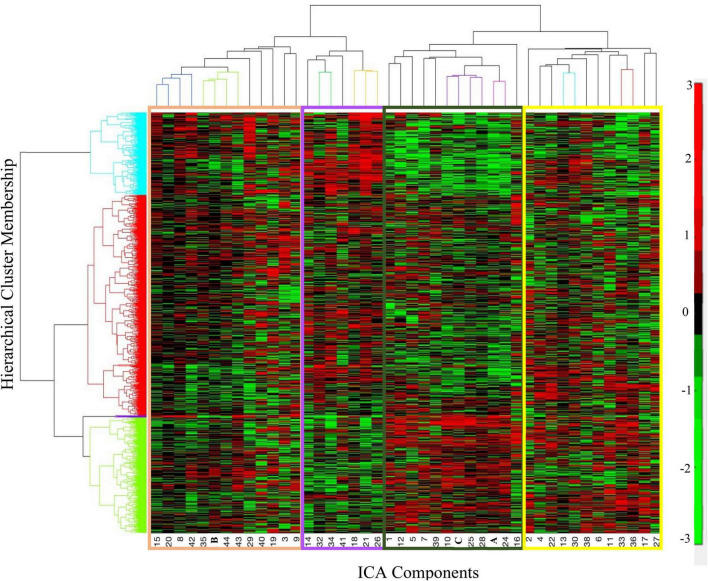
Hierarchical cluster analysis of independent component analysis (ICA) loading coefficients resulted in four clusters of participants (Y-axis, rows). The y-axis shows the cluster membership, the X-axis shows the ordering of the ICA components. The organization of the components along the X-axis is color-coordinated with Table 5. The tan cluster is largely comprised of posterior cortical regions. The purple cluster is largely negatively weighted on the cerebellar regions. The dark green cluster includes Components A and C as well as cerebellar and subcortical components. The yellow cluster includes most of the frontal as well as cortical components from every other lobe. See Table 5 for more details on the peak locations of each component. In the color bar, red represents positive loading coefficients and green represents negative loading coefficients.

In the hierarchical cluster analysis of the 44 gray matter components’ loading coefficients, four clusters were identified. The four clusters appear to fall along the divisions of cortical regions, subcortical regions, and two cerebellar regions (see Figure 5 and Table 5 for cluster membership). There were no significant associations between membership in any of the four ICA clusters and the six PANSS clusters [χ^2^(15) = 16.79, *p* = 0.331]; any of the PANSS subscale scores (PANSS positive [χ^2^(52) = 37.87, *p* = 0.929]; PANSS negative [χ^2^(58) = 57.20, *p* = 0.505]; PANSS general [χ^2^(78) = 93.33, *p* = 0.114] or diagnostic group membership [χ^2^(2) = 1.22, *p* = 0.543]. Supplementary Figure 1 details all the ICA components in the cluster groupings.

**TABLE 5:** Description of the peak locations of each independent component analysis (ICA) component.

Component	Positive loadings	Negative loadings
15	Putamen L/R	
20	Calcarine R/L	
8	Vermis; cerebellum 9	Caudate R/L; putamen L/R; thalamus L/R
42	Cerebellum L/R	
35		Superior temporal gyrus
**B**	Cerebellar hemisphere; vermis L/R	
44	Thalamus L/R	
43	Parietal sup L	Thalamus L/R
29	Cingulum	Frontal mid L/R
40	Insula L/R	Fusiform L/R
19	Angular L/R; temporal sup R	Parietal inf L/R; angular L
3	Calcarine L/R	Cuneus L/R; occipital Sup R; precuneus R
9	Occipital sup R; occipital mid L	Calcarine L/R; precuneus L/R; posterior Cingulum
14		Cerebellum L; Crus I L/R; cerebellum 7b
32		Precentral L/R
34		Supp motor area R; precuneus
41	Frontal mid L	Vermis 4 5; cingulum post L/R; lingual L/R
18		Crus II L/R
26	Temporal inferior gyrus L/R; fusiform L/R	Temporal inferior gyrus L/R; fusiform L/R
1	Crus II L/R	
12	Cerebellum/vermis	
5	Cerebellum 9 L/R	
7	Ventricles	
39	Fusiform L/R; lingual L; Crus I L/R	Cerebellum 6 L/R; Crus I L/R; vermis 7
10	Cerebellum Crus I L/R; calcarine L	
**C**	Temporal poles L/R	
25	Cerebellum 8 L/R	Lingual R; calcarine R
28	Rectus L/R	Cerebellum 8 L; fusiform L
**A**	Insula L/R; cingulate gyrus L/R; temporal pole sup L/R; temporal sup R	
24	Frontal mid L/R; frontal inf Tri L/R	
16	Occipital mid L; parietal inf L/R; angular L/R; temporal mid L/R	Temporal mid L/R; angular L/R; occipital sup L/R
2	Frontal inf tri L/R; frontal sup L/R	Frontal mid L/R; insula L/R
4	Temporal sup L; insula L	Temporal mid L; temporal sup R
22	Precentral L/R	Frontal inf oper L/R; frontal mid L/R; precentral L/R
13	Cuneus L; insula R; angular R; temporal mid R; temporal sup R; temporal inf R	Temporal mid R; temporal sup R
30	Temporal mid L/R; parietal inf L; cuneus L/R; frontal inf oper R; supramarginal R	Rolandic oper L/R; temporal pole sup L/R; Calcarine L/R; Rectus L/R; insula L/R
38	Parietal inf lobule L/R; angular L/R	Cuneus L/R; precuneus L/R
6	Calcarine L; Crus II L; temporal inf R; Crus I R; lingual L	Crus II R; lingual R; calcarine R
11	Frontal inf tri L/R; frontal sup L; frontal mid L/R	Frontal mid L/R; frontal inf tri L/R; frontal inf oper L/R
33	Calcarine L/R	Parietal inf L; frontal Mid L/R; supra marginal R; angular R; frontal sup L/R
36	Parietal sup L/R; parietal inf R	
17	Occipital mid L; temporal mid R; angular R	Occipital mid L/R; occipital sup L/R
27	Temporal inf L/R; temporal mid L/R; angular L/R	Temporal mid L/R; occipital mid L

Table is in matching order to the hierarchical clustering of the ICA depicted in Figure 5. The color separation shows the four (4) clusters of the hierarchical cluster (tan, purple, green, and yellow). Description of the locations of the peak(s) identified within each component. L, left; R, right; peak regions identified using the aal.nii template.

### N-way biclustering

**FIGURE 6:**
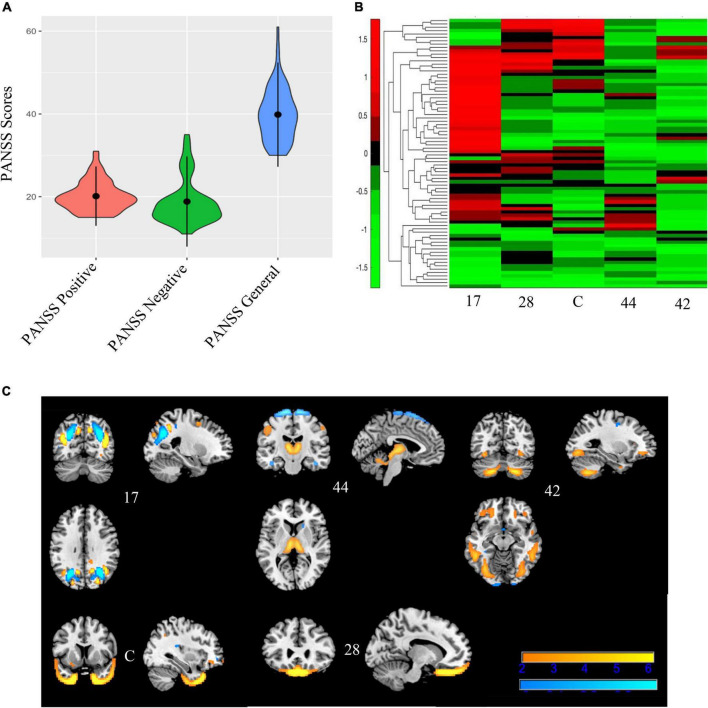
Bicluster 1: PANSS scores, heat map of loadings of independent component analysis (ICA) components, and ICA components. Bicluster 1 (*N* = 80) had more severe scores on all three PANSS subscales **(A)** than the rest of the sample. This bicluster included 5 ICA components (17, 44, 42, C, and 28) representing the occipital lobes, rectus, cerebellum, and the temporal poles that were generally low in the loading coefficients **(B)**. Images in **(C)** 6C are thresholded at |2.5|. Warm colors represent positive values and cool colors represent negative values.

**FIGURE 7:**
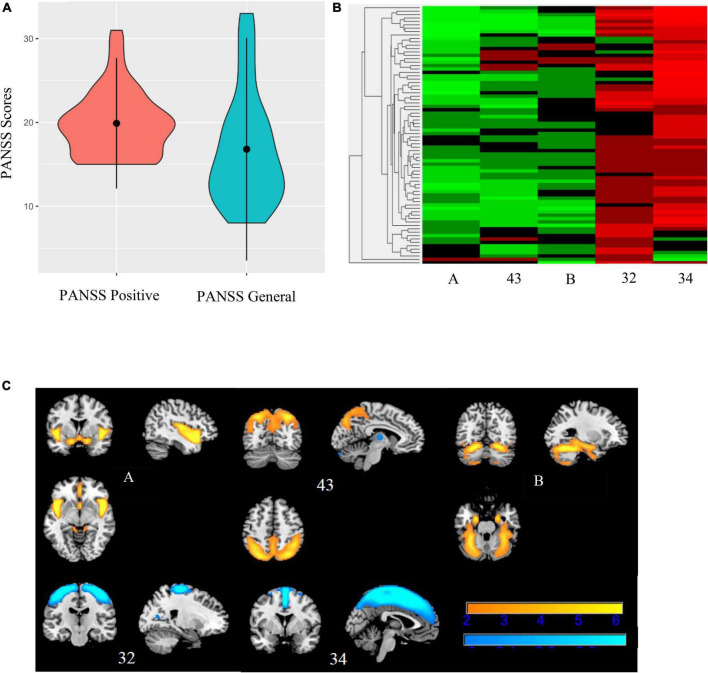
Bicluster 2: PANSS scores, heat map of loadings of independent component analysis (ICA) components, and ICA components. Bicluster 2 (*N* = 76) consisted of more severe PANSS positive and PANSS general scores **(A)** and 5 ICA components (A, 43, B, 32, and 34) representing the insula, cerebellum, and supplemental motor and motor cortices **(B)**. Images in **(C)** 7C are thresholded at |2.5|. Warm colors represent positive values and cool colors represent negative values.

**FIGURE 8:**
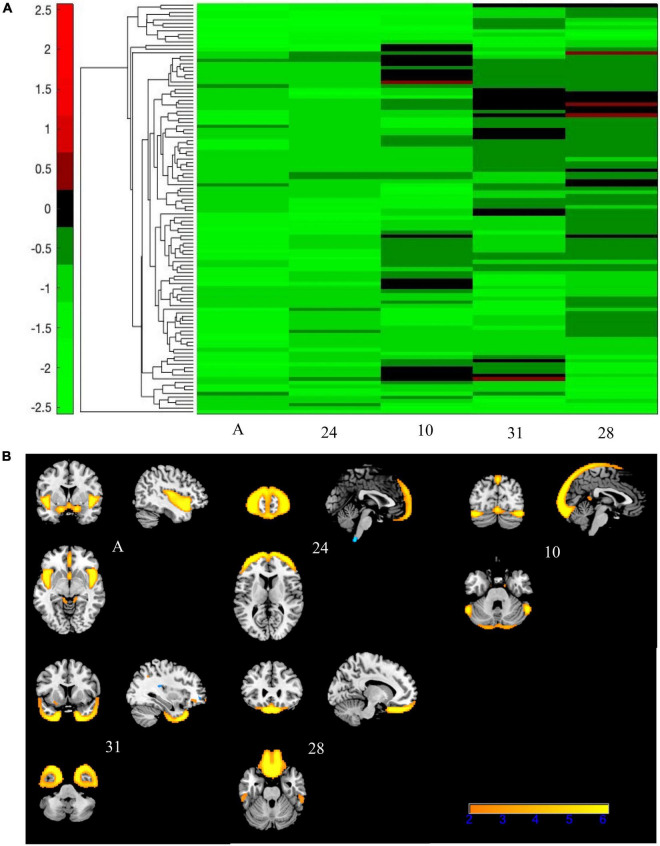
Bicluster 3: heat map of loadings of independent component analysis (ICA) components and related ICA components. Bicluster C (*N* = 112) was comprised of five ICA components (A, 24, 10, 31, and 28) shown above that had mostly negative loading coefficients **(A)**. There were no PANSS subscales associated with bicluster C. Images in **(B)** 8B are thresholded at |2.5|. Warm colors represent positive values.

The bi-cluster analyses identified nine bi-clusters within the data matrix. Of those, three bi-clusters had high reliability (*freq* > 3). Spatial maps of the components included in these clusters, heat maps indicating the loading coefficients of each of these spatial maps, and heat maps of the related PANSS subscale scores are shown in Figures 6–8, respectively.

Bicluster 1 included 80 individuals (14 BP; 66 SZ) and clustered on all three PANSS subscales (positive, negative, and general) and five ICA components. See Figure 6 for more details. The average age for bicluster 1 was 36.35 years old (SD = 11.08) and there were 50 male participants (62.50%). Bicluster 1 had more severe PANSS scores than the rest on all three subscales: positive mean = 20.14 (SD = 3.59); negative mean = 18.84 (SD = 5.44); general mean = 39.84 (SD = 6.30). The five ICA components showed varying degrees of alterations in the gray matter of the thalamus, cerebellum, occipital lobes, rectus, and the temporal poles. Of these, only two components (28 and C) showed group differences between healthy volunteers and schizophrenia.

Bicluster 2 included 76 individuals (17 BP; 59 SZ) and clustered on PANSS positive scores, PANSS general scores, and five ICA components. See Figure 7 for more details. The average for bicluster 2 was 37.09 years old (SD = 11.00) and there were 47 male participants (61.84%). Bicluster 2 had, on average, more severe PANSS positive (mean = 19.89; SD = 3.90) and lower PANSS general (mean = 16.80; SD = 6.64) scores than the rest of the sample. All five ICA components also showed lower gray matter concentration (cluster peaks identified in the insula, cerebellum, and supplemental motor and motor cortices) when compared to the rest of the sample. Of these five components, two components (A and B, described above) showed diagnostic group difference for both SZ and BP (HV > SZ; HV > BP), and one showed a significant group difference between HV and SZ.

Bicluster 3 consisted of 112 participants (32 BP; 80 SZ) and five ICA components but no PANSS subscales. See Figure 8 for more details. The average age for bicluster 3 was 36.66 years old (SD = 11.18) and there were 67 male participants (59.82%). All five ICA components within bicluster 3 showed significant group differences (A and 24: HV > SZ, HV > BP; 10: HV > SZ; C: HV > SZ, BP > SZ; 28: HV > SZ). Bicluster 3 had reduced gray matter concentration in the frontal middle gyrus, frontal inferior gyrus, insula, cerebellar Crus I, rectus, and fusiform gyrus.

To gauge consistency between the hierarchical and biclustering methods, we asked whether the participant groups identified in each overlapped. Of the four hierarchical clusters based on gray matter loading coefficients only one cluster had the greatest amount of overlap with all the biclusters (hierarchical cluster shown in light blue, Figure 4). There were 69 (61.61%) individuals from bicluster 3, 37 (48.68%) individuals from bicluster 2, and 9 (11.25%) from bicluster 1 in this cluster. There were eight individuals who were present in all three biclusters. We would not expect to see exact overlap between the clustering as the PANSS scores are influencing the identification of reliable groups in the n-way biclustering and hierarchical clustering was used to examine either the ICA components or PANSS symptoms scores (two separate models).

We completed subsequent *post hoc* analyses with individuals for whom we had chlorpromazine equivalents (CPZ; *N* = 381) and found that component A was unaffected by CPZ scores, but that component B was significantly negatively related to CPZ scores (*r* = −0.129, *p* = 0.012). In other words, individuals with high CPZ scores had less gray matter in the cerebellum. Similarly, we completed subsequent analyses with duration of illness (DOI) and although we were limited in the amount of participant information (*N* = 640) and we found no changes to our significant association in individuals with BP but with individuals with SZ, DOI was significantly negatively related to both component A (*r* = −0.165, *p* = 5.58E-5) and B (*r* = −0.101, *p* = 0.014). In other words, individuals with longer durations of illness had less gray matter in both the insula/cingulum component and the cerebellum component.

## Discussion

We examined patterns of gray matter alterations in SZ, BP, and HV. We also examined the symptom profiles of the diagnostic groups to parse out unique subgroups. We evaluated outcomes of ICA and symptom pattern analyses to link gray matter patterns with unique symptom profiles.

The ICA of gray matter variations demonstrated that individuals with SZ and BP share similar patterns of structural deficits. We identified two components that showed significant group differences between HV and BP, and HV and SZ. Component A showed less gray matter bilaterally in the insula and cingulate in both diagnostic groups compared to HV. This pattern has been previously identified robustly in the schizophrenia literature (Segall et al., 2009; Gupta et al., 2015; Meda et al., 2015; Jiang et al., 2021). Component B showed less gray matter bilaterally in the cerebellum in both diagnostic groups compared to HV. The cerebellar GM reductions supports previous literature that linked these losses to cognition (e.g., long-term and working memory) and symptoms (Moberget et al., 2018, 2019). Our results are in line with previous literature that shows a relationship between psychosis and the frontotemporal cortices in BP (Ivleva et al., 2013) and the cerebellum in adolescents, regardless of SZ or BP diagnosis (Moberget et al., 2019).

Component C showed significantly lower gray matter bilaterally in the fusiform gyrus/temporal pole of SZ vs. between BP where BP were not different from HV. The fusiform gyrus is a key region for sociality-related high-level vision (e.g., face perception) (Lee C. U. et al., 2002; Rangarajan et al., 2014). While our results may have been a function of having a smaller BP sample, this component is unique across the other components in showing no difference between BP and HV, while showing a strong reduction in SZ compared to BP. This result supports previous literature that found small effect sizes in this region in individuals with BP (Murray et al., 2004; Bora, 2015; Hibar et al., 2018; de Zwarte et al., 2019). The fusiform gyrus was also an area of discrepancy between individuals with SZ and individuals with schizotypy with the region being only reduced in SZ (Dickey et al., 2003; Onitsuka et al., 2003; Takahashi et al., 2006). We hypothesize that these results may indicate that the fusiform gyrus/temporal pole is a region strongly related to SZ but largely unaffected in other psychiatric disorders (e.g., BP, schizophrenia spectrum disorders).

Individuals with SZ and BP presented with similar symptom profiles, where individuals with SZ had more severe symptoms in the three PANSS subscale scores. ICA components identified by the N-way biclustering did not allow for diagnostic distinction between participants as it relates to structural alterations. The hierarchical clustering of the PANSS subscales produced clusters that were unique in their individual subscale totals, but there were no significant relationships between any of the clusters and the ICA components, indicating that the clusters were not identifying symptom profiles that related to a unique structural correlate. Of the six clusters, one had significantly more BP participants than the others and one had significantly fewer BP participants than the others. Similar to our results, Clementz et al. (2016) found a homogeneous subgroup with more severe clinical symptoms (e.g., negative symptoms and social functioning) that was majority SZ (56%) (Clementz et al., 2016, 2020). Both these previous findings and those discussed here support the main difference between SZ and BP being the severity of symptoms with no discerning differences in brain structure.

A limitation of the hierarchical cluster analysis is that the entirety of the data matrix is utilized for cluster profiling. We hypothesized that additional symptom profiles may appear if the analysis was not required to include all the PANSS subscales. Therefore, we explored the potential relationship between the PANSS symptom profiles and the ICA components by adding both to a n-way biclustering analysis. A strength of the biclustering approach is that the data matrix is examined using every possible combination within the data, and therefore, submatrices or clusters may arise that do not include all participants, subscales, or components.

Our results found three biclusters; Bicluster 1 included all three subscales of the PANSS, Bicluster 2 included PANSS positive and PANSS general, and Bicluster 3 did not include any PANSS subscales. Bicluster 1 (*N* = 80) included both individuals with SZ and individuals with BP who had more severe symptom scores on all three PANSS subscales. The five ICA components that were identified in bicluster 1 included peaks in the occipital lobe, cerebellum, bilateral temporal poles, thalamus, and bilateral rectus. However, the directionality of these peaks was not uniform (see Figure 5) across participants. Therefore, we conclude that, this cluster identifies a subgroup in which more severe positive, negative, and general psychopathology symptoms are related to less gray matter in the cerebellum, thalamus, and bilateral rectus; but we also note that there is inconsistency in the directionality of the gray matter alteration in the bilateral temporal poles and occipital lobes. These inconsistencies may be explained by the range of symptom presentations included in the bicluster. More severe PANSS positive scores have been associated with *regional* cortical thinning whereas more severe PANSS negative scores were associated with *global* cortical thinning (van Erp et al., 2018). Regardless, this bicluster represents a group of people that, regardless of diagnosis, share both a pattern of low gray matter concentration and severe total symptom profiles.

Recently, the rectus has been shown to be associated with individuals with high genetic risk for either SZ, BP, or psychosis (Luna et al., 2022). As mentioned in the previous section, component C (part of this bicluster) has also been studied extensively in the schizophrenia spectrum literature (Takahashi et al., 2006). Previous literature has also found that poor facial recognition is associated with the genetic risk for SZ (Martin et al., 2020). Therefore, this bicluster may give insight into the genetic risk of developing psychosis, but further research is needed.

Bicluster 2 (*N* = 76) included both individuals with SZ and individuals with BP who had more severe PANSS positive and lower PANSS general scores than the rest of the sample. All five ICA components identified in this bicluster indicated less gray matter concentration than the rest of the sample. In other words, this bicluster showed a negative relationship with more severe PANSS positive scores, low PANSS general scores, and less gray matter in all five ICA components. Component A and Component B which each showed significant reductions in both diagnostic groups, SZ and BP, were both included in bicluster 2. In addition, there were three other components (involving the precentral gyrus, thalamus, left parietal supplemental gyrus, and right supplemental motor region) where bicluster 2 had less gray matter concentration than the rest of the sample. And as mentioned above, three of these components identified significant diagnostic group differences. Therefore, bicluster 2 indicates that more severe positive symptoms and less prominent general psychopathology symptoms are related to less gray matter in the cerebellum, insula, and other subcortical regions.

Bicluster 3 (*N* = 112) identified a subset of the sample that presents with a unique structural set of patterns but there are no clinical features (symptom scores, age, gender, and site) that distinguish this cluster from the rest of the sample. Given the directionality of gray matter alteration, we hypothesize that this bicluster may be identifying individuals with similar cortical measures (e.g., smaller brain volumes overall) and not necessarily unique clinical presentations. In addition, one component showed lower gray matter concentration in the rectus gyrus, which has been associated with genetic risk for SZ, BP, and psychosis (Luna et al., 2022). It is possible that this bicluster is mapping onto a genetic profile instead of a clinical profile. However, we caution that further research is needed to confirm the role of this bicluster in genetic risk for psychiatric disorders.

The addition of n-way biclustering to the analyses allowed for identification of reliable grouping of the sample based on both the symptom profiles and the gray matter patterns. These results included unique, homogeneous subclusters that were not identified in the ICA results or the hierarchical cluster analyses. The bicluster approach identified anatomic substrates that related to different symptoms profiles in both SZ and BP, supporting a dimensional view of these disorders.

### Limitations

There are a few limitations of this study. The number of BP individuals (*N* = 301) across all the sites was significantly less than the number of SZ individuals (*N* = 1217). We acknowledge that this imbalance in diagnostic groups may have masked some of the BP specific results, especially in the ICA components. However, *post hoc* analyses of group differences in HV v. BP and BP v. SZ showed significant differences and strong effect sizes. Although there is an imbalance in our diagnostic group sizes, we were still able to identify some valid representations of gray matter alterations in BP. The imbalance in the number of BP and SZ participants may also have contributed to the significant site effects that were observed in the PANSS subscales as not every site had the same distribution. We also acknowledge that our findings can be confounded by medication effects. We do not have medication information for every individual in the sample and we cannot conclude that our results are not at least somewhat driven by medication differences. There were also a large number of sites (*N* = 29) included in the study. Site effects were addressed in the preprocessing pipelines and the subsequent analyses, but site-specific effects may still occur as detailed in Supplementary Table 2.

Another limitation of this study is the utilization of the PANSS subscales for symptom profiling. Although the PANSS is a gold-standard assessment for SZ and widely utilized in BP, this assessment is not all-encompassing of the multitude of symptoms presenting in both schizophrenia and bipolar disorder over time. Especially for individuals with BP, there may be some symptom presentations that fluctuate with mood or psychosis and therefore, cross-sectional assessments may not capture all symptoms. Further research may want to examine additional scales (e.g., Montgomery-Aberg Depression Rating Scale) that capture other symptoms that may round out the symptom profile as previous studies have done with their subtyping of psychosis-related disorders (Clementz et al., 2020) and scales that examine these symptoms over the lifetime (as opposed to recent or the previous 2 weeks).

## Conclusion

Our study examined multivariate relationships between the symptom profiles and gray matter patterns of individuals with SZ and individuals with BP. Our results identified one gray matter pattern that differed between SZ and BP. However, this pattern (less gray matter concentration in the temporal poles) was not significantly different between individuals with BP and healthy volunteers. Therefore, we conclude that this region is much less affected, if at all, in BP. The remainder of our results indicate that SZ and BP lie along an extensive spectrum of symptoms and brain correlates but there are no clear distinctions between the disorders in these areas.

However, we note that there are still meaningful distinctions between the disorders. The category of symptoms differ, the prognosis and diagnostic timeline often differ, and most importantly, the prescribed treatments often differ between individuals with SZ and individuals with BP. Overall, we conclude that the cortical alterations seen in individuals with SZ and individuals with BP trend together and are not significantly different from one another; similar patterns of gray matter loss particularly in the cerebellum and thalamus or insula appear to cluster with more severe symptoms. All components identified in this study showed the same direction of gray matter concentration (SZ < BP < HV). Based on these findings, it appears that BP and SZ may track along the same spectrum, with individuals with BP having less severe cortical alterations and less severe symptom profiles when compared to individuals with SZ, but without a clear distinction in cortical alterations between these disorders.

## Data availability statement

The data analyzed in this study is subject to the following licenses/restrictions: Some of the datasets are already publicly available (see citations in manuscript). Some are still privately owned. Requests to access these datasets should be directed to http://fcon_1000.projects.nitrc.org/indi/retro/cobre.html.

## Author contributions

WJ, KR-M, and JT: conceptualization. WJ, KR-M, JE, and MR: methodology. KR-M and JE: writing—original draft preparation. NP-B, VC, TE, SE, IA, EJ, OA, LWe, LWa, GP, DG, EH, RB, PK, AV, AM, CT, and JL: data collection and curation. JT: supervision. All authors writing—review and editing, read, and agreed to the published version of the manuscript.

## Footnotes

1
http://www.fil.ion.ucl.ac.uk/spm/software/spm12/

2
https://trendscenter.org/software/gift/

3
https://github.com/trendscenter/gift
