# Supplementary material for: Clinical and cortical similarities identified between bipolar disorder I and schizophrenia: A multivariate approach
Source: Front Hum Neurosci. 2022 Nov 10;16:1001692. doi: 10.3389/fnhum.2022.1001692 (PMC9684186; doi:10.3389/fnhum.2022.1001692)

| **Supplemental Table 1.** All Nonsignificant Group Differences across ICA Components. | | | |
| --- | --- | --- | --- |
|  | **Component Number** | ***F*** | ***p* value** |
| **HV v BP v SZ** | 1 | 2.538 | .080 |
|  | 2 | 1.729 | .178 |
|  | 3 | 3.524 | .030 |
|  | 4 | 0.850 | .428 |
|  | 6 | 3.605 | .027 |
|  | 9 | 1.182 | .307 |
|  | 11 | 4.202 | .015 |
|  | 12 | 0.444 | .642 |
|  | 13 | 6.041 | .002 |
|  | 14 | 0.745 | .475 |
|  | 15 | 4.163 | .016 |
|  | 16 | 0.389 | .678 |
|  | 17 | 2.590 | .075 |
|  | 19 | 1.664 | .190 |
|  | 21^*^ | 67.172 | 2.822E-29 |
|  | 22 | 4.279 | .014 |
|  | 25 | 4.131 | .016 |
|  | 29 | 4.432 | .012 |
|  | 32 | 1.645 | .193 |
|  | 38 | 5.035 | .007 |
|  | 39 | 0.374 | .688 |
|  | 40 | 0.089 | .915 |
|  | 41 | 1.475 | .229 |
|  | 42 | 2.018 | .133 |
|  | 43 | 1.906 | .149 |
|  | 44 | 2.408 | .090 |

Between group differences for all ICA components not reported in Table 3. Statistical results for all group comparisons (HV v BP; HV v SZ; BP v SZ) were thresholded at α < .001136 with Bonferroni (*p* = .05 / 40) correction. ^*^Component 21 was noted as significant but was determined to be most likely related to motion (see Table 5 for peak description).

**Supplemental Table 2.** Components with Significant Covariate Effects.

Supplemental Table 2 contains the components that had significant effects for either site, age, and/or sex. Standardized betas are listed. All *p* values are uncorrected.

| Component | Covariate | β | *t* | *p* |
| --- | --- | --- | --- | --- |
| 19 | Site | .063 | 3.492 | 4.86E-4 |
|  | Diagnosis | .037 | 2.017 | .044 |
|  | Age | .005 | 0.263 | .792 |
|  | Sex | -.002 | -0.124 | .901 |
| A | Site | -.015 | -0.843 | .399 |
|  | Diagnosis | -.266 | -15.030 | 2.594E-49 |
|  | Sex | -.038 | -2.133 | .033 |
|  | Age | -.003 | -0.148 | .883 |


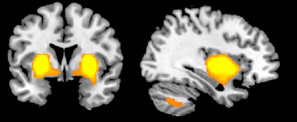


15


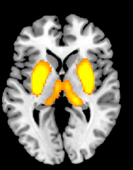

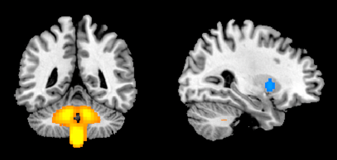

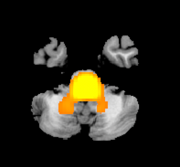


8


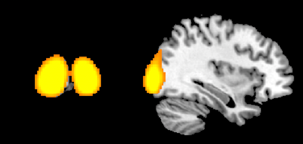

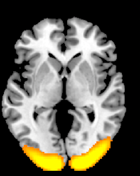


20

**Supplemental Figure 1.** All ICA Components Sectioned into the Four Clusters Membership.

15

20

8

15


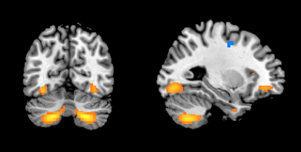


42


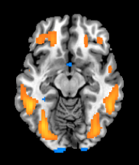

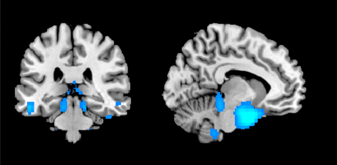

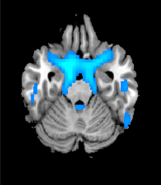


35


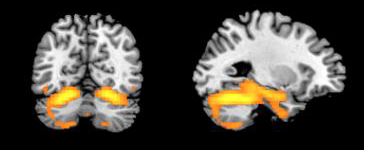

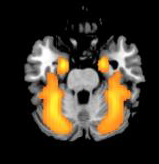


B


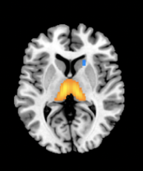

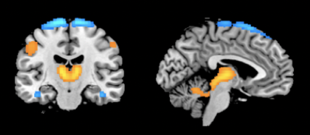


44


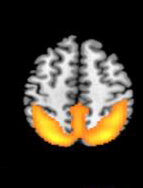

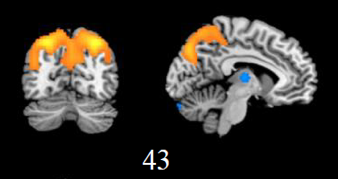


43


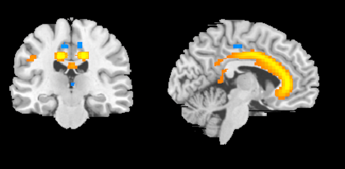

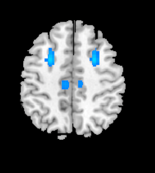


29


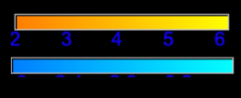


2 3 4 5 6

-2 -3 -4 -5 -6


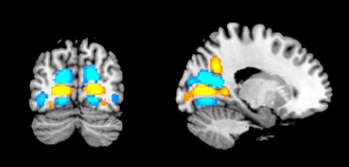

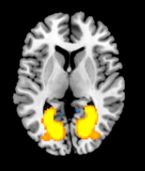


3


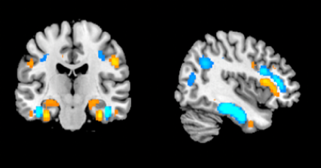


40


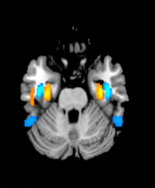

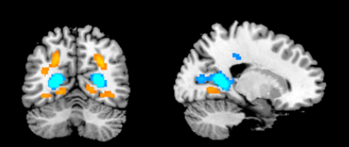

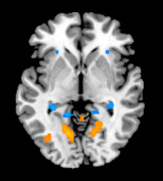


9


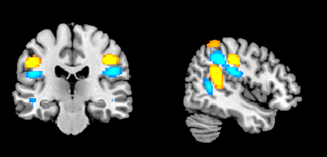

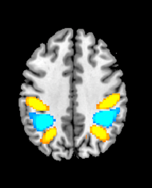


19

The 13 ICA components in the first cluster of the cluster analysis. This cluster (referred to as the tan cluster in Figure 5 and Table 5) is comprised of mostly posterior cortical regions. Images are thresholded at |2.5|.


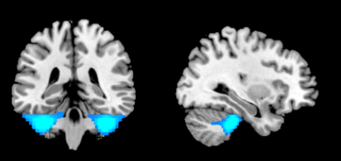

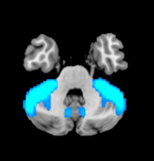


14


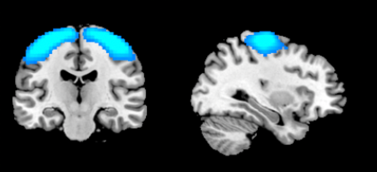

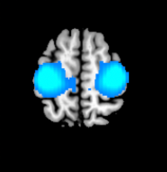


32


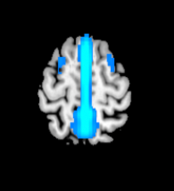

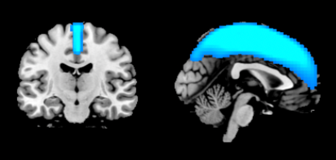


34


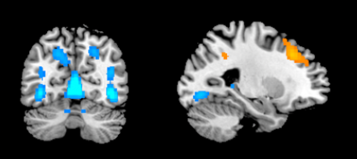

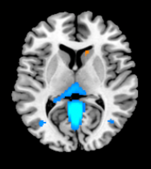


41


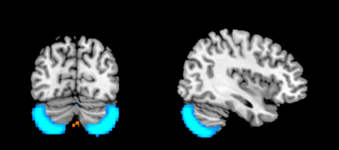

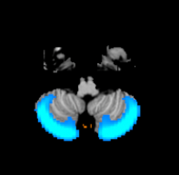


18


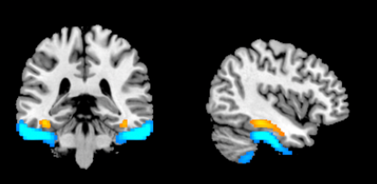

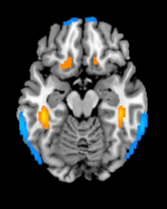


26


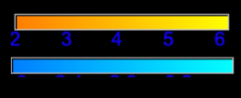


2 3 4 5 6

-2 -3 -4 -5 -6


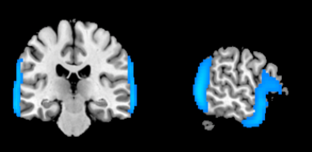

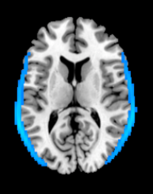


21

The seven ICA components in the second cluster of the cluster analysis. This cluster (referred to as the purple cluster in Figure 5 and Table 5) is comprised of mostly negatively and cerebellar regions. Images are thresholded at |2.5|.


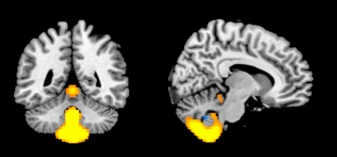

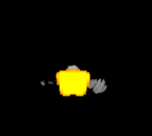


5


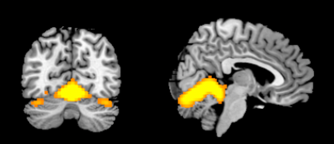

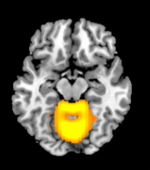


12


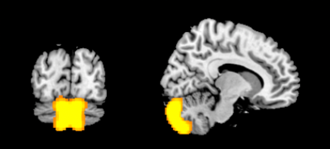

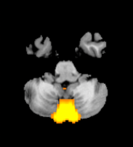


1


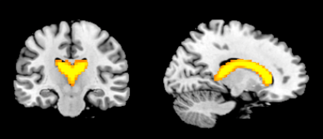

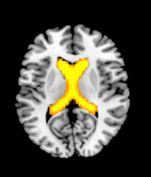


7


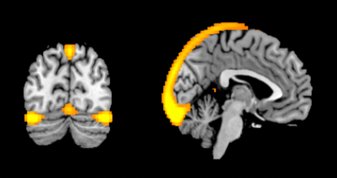

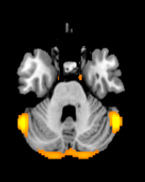


10


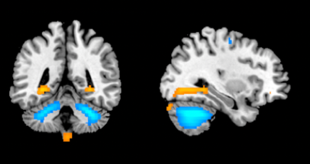


39


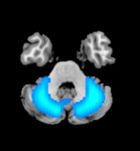

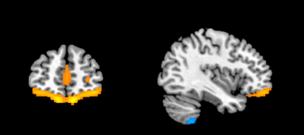

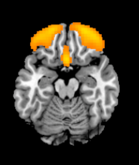


28


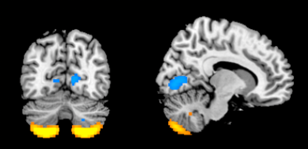

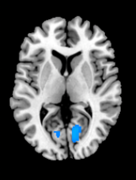


25


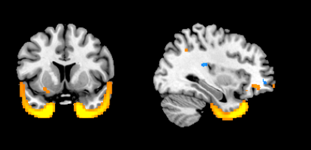

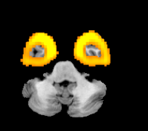


C


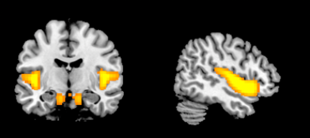

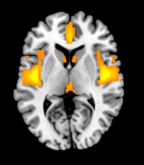


A


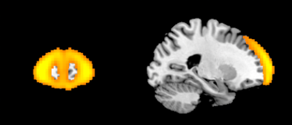

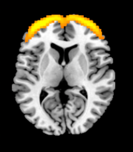


24


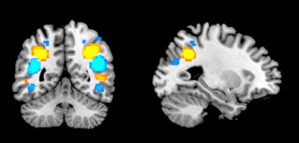

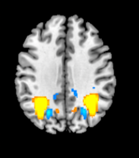


16


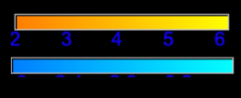


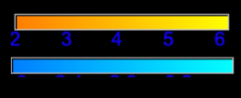


2 3 4 5 6

2 3 4 5 6

-2 -3 -4 -5 -6

-2 -3 -4 -5 -6

The twelve ICA components in the third cluster of the cluster analysis. This cluster (referred to as the dark green cluster in Figure 5 and Table 5) is comprised of Components A and C as well as cerebellar and subcortical components. Images are thresholded at |2.5|.


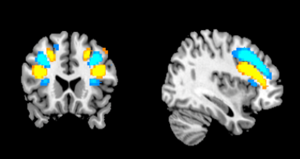

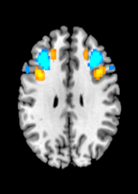


2


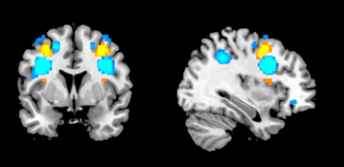

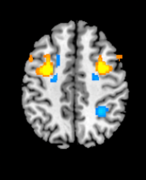


22


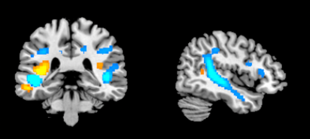

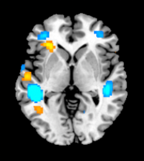


4


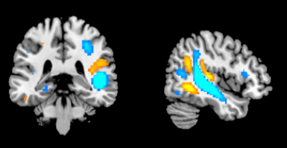

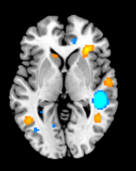


13


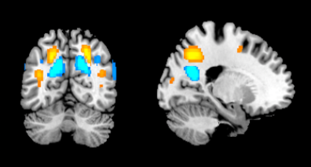

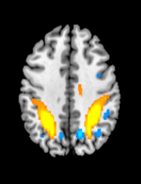


38


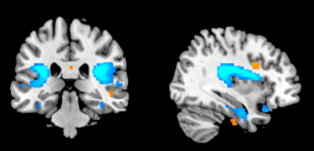

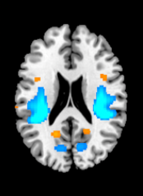


30


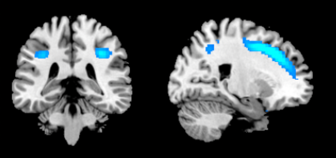

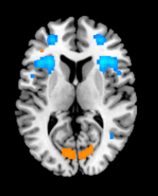


33


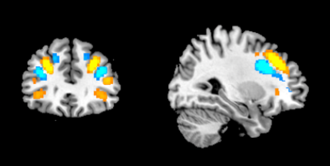

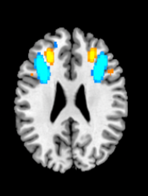


11


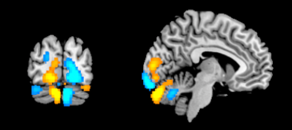

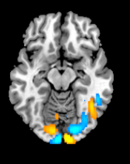


6


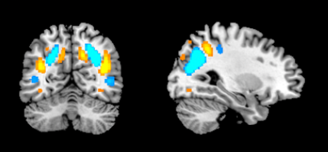

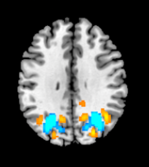


17


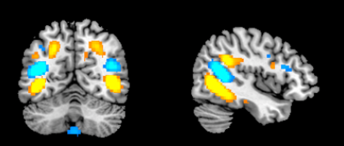

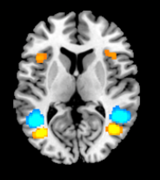


27


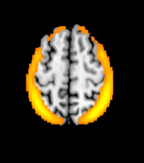

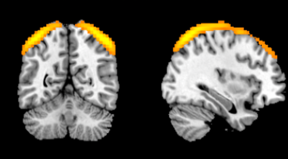


36


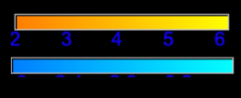


2 3 4 5 6

-2 -3 -4 -5 -6

The twelve ICA components in the fourth cluster of the cluster analysis. This cluster (referred to as the yellow cluster in Figure 5 and Table 5) is comprised of most of the frontal as well as side-by-side positive and negative loadings of cortical components from every other lobe. Images are thresholded at |2.5|.

**Supplemental Figure 2.** PANSS Spread Across Clusters.

The PANSS positive, negative, and general scores across each cluster (N = 6) from the PANSS hierarchical cluster analysis. None of the clusters were significantly associated with ICA components.


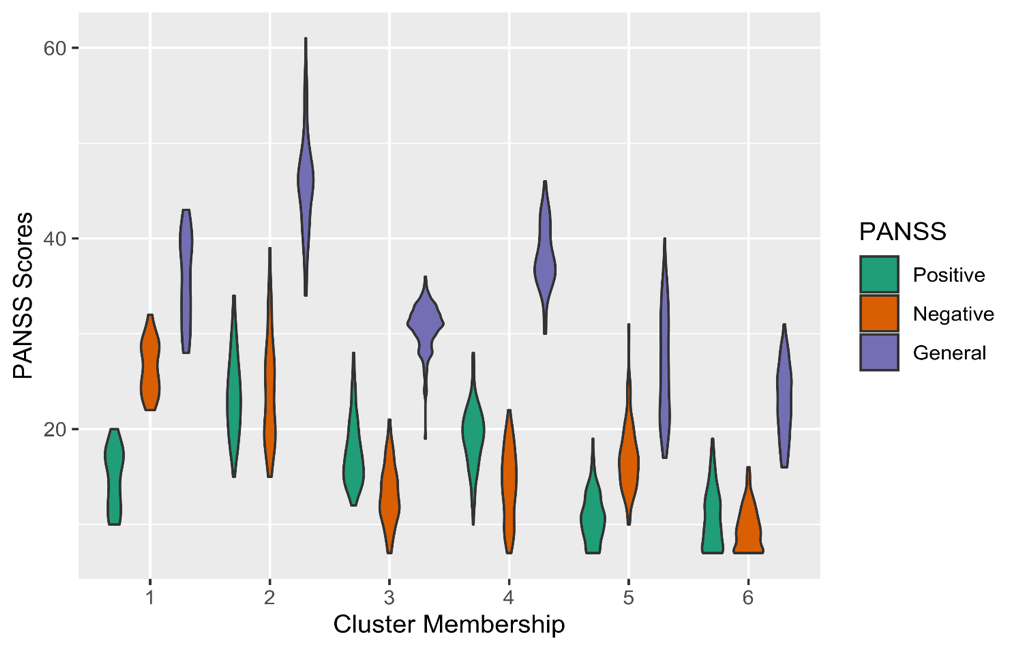

Supplement: Supplementary file 1 [file Data_Sheet_1.docx]
